# C–H Labeling with [^18^F]Fluoride: An Emerging Methodology in Radiochemistry

**DOI:** 10.1021/acscentsci.4c00997

**Published:** 2024-08-23

**Authors:** Jay S. Wright, Liam S. Sharninghausen, Alex Lapsys, Melanie S. Sanford, Peter J. H. Scott

**Affiliations:** † Department of Radiology, University of Michigan, Ann Arbor, Michigan 48109, United States; ‡ Department of Chemistry, University of Michigan, Ann Arbor, Michigan 48109, United States

## Abstract

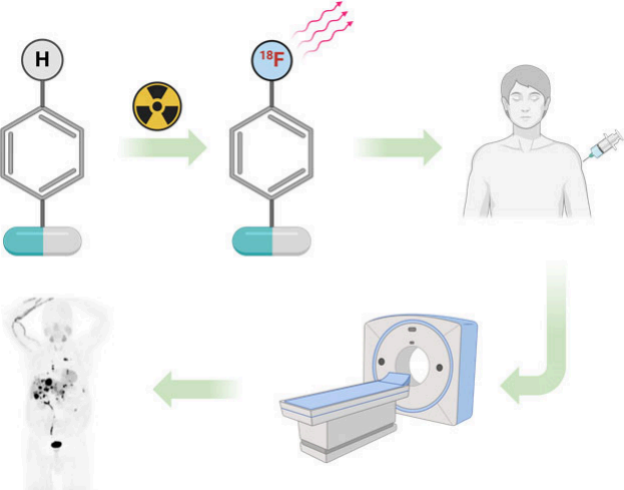

Fluorine-18 is the most routinely employed radioisotope for positron emission tomography, a dynamic nuclear imaging modality. The radiolabeling of C–H bonds is an attractive method for installing fluorine-18 into organic molecules since it can preclude the cumbersome prefunctionalization of requisite precursors. Although electrophilic “F^+^” reagents (e.g., [^18^F]F_2_) are effective for C–H radiolabeling, state-of-the-art methodologies predominantly leverage high molar activity nucleophilic [^18^F]fluoride sources (e.g., [^18^F]KF) with substantial (pre)clinical advantages. Reflecting this, multiple nucleophilic C–H radiolabeling techniques of high utility have been disclosed over the past decade. However, the adoption of (pre)clinical C–H radiolabeling has been slow, and PET imaging agents are still routinely prepared via methods that, despite a high level of practicality, are limited in scope (e.g., S_N_Ar, S_N_2 radiofluorinations). By addressing the drawbacks inherent to these strategies, C–H radiofluorination and radiofluoroalkylation carry the potential to complement and supersede state-of-the-art labeling methods, facilitating the expedited production of PET agents used in disease staging and drug development. In this Outlook, we showcase recent C–H labeling developments with fluorine-18 and discuss the merits, potential, and barriers to adoption in (pre)clinical settings. In addition, we highlight trends, challenges, and directions in this emerging field of study.

## Abstract 2

C−H labeling methods for PET rapidly incorporate fluorine-18 using stable precursors with minimal prefunctionalizations. This improves access to radiomedicines, enhancing bioimaging and drug discovery.

## 1 Introduction

**Figure 1:**
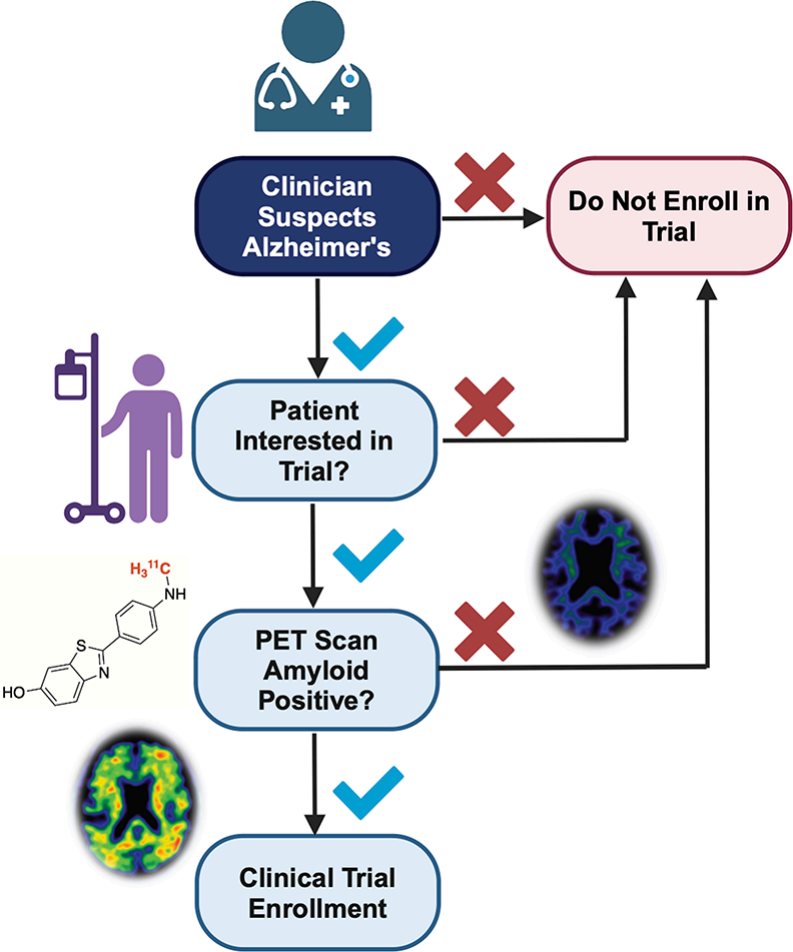
Utility of [^11^C]PiB positron emission tomography in human clinical trials toward the development of Alzheimer’s therapeutics.

In a positron emission tomography (PET) scan, the detection of two coincident γ-ray photons emitted upon the annihilation of a β^+^ particle originating from radioactive decay yields dynamic information about the localization, distribution, and accumulation of a radiopharmaceutical *in vivo*. Therefore, radiolabeled chemical entities, such as small organic molecules, are commonly utilized by imaging scientists for (pre)clinical PET to noninvasively study, stage, and detect diseases and disorders spanning nearly all (sub)disciplines of medicine. Furthermore, PET can support drug development by evaluating drug-target engagements, dosage requirements, and patient population enrichment in clinical trials. For example, [^11^C]Pittsburgh Compound B ([^11^C]PiB) is one of multiple amyloid imaging agents that has been used to assess the therapeutic efficacy of drug candidates in treating Alzheimer’s disease (Figure 1).^1^ Carbon-11 labeled small organic molecules are an important class of β^+^ PET imaging agent particularly for preclinical purposes, although the longer half-life of fluorine-18 (*t*_1/2_ = 109.8 min vs 20.4 min) is more attractive for off-site transport and commercialization. Accordingly, around two million PET scans are conducted in the USA annually, with fluorine-18 as the current radionuclide of choice for these analyses owing to optimal imaging properties in small organic molecules.^2^ For example, the combination of a simple emission signature (97% β^+^ decay), ready availability in multi-Curie quantities from small medical cyclotrons (permitting on- and off-site usage), and a high prevalence of marketed fluorine-19-containing pharmaceuticals render fluorine-18 an attractive radionuclide in the design and production of PET imaging agents.^3,4^ In the United States, recent approvals by the Food and Drug Administration (FDA) of new fluorine-18 imaging agents in conjunction with improved coverage options from the Centers for Medicare and Medicaid Services (CMS) have contributed to an increased demand for medical PET imaging scans and, by extension, the expedited discovery and preparation of fluorine-18 nuclear medicines.^5^ Among the many fluorine-18 motifs, including [^18^F]fluoride salts, Al–^18^F, B–^18^F, S–^18^F, and Si–^18^F bonds, C–^18^F bonds are the most widely used owing to a high utility and ubiquity of carbon in tracer scaffolds.^6^ Therefore, the potential and utility of PET in industrial, healthcare, and academic settings are inextricably linked with the availability of radiosynthetic methods for efficiently and selectively constructing C–^18^F bonds with optimal *in vivo* stability. A wealth of research on the reactivity of fluorine-19 is available to radiochemists to support this. However, despite many parallels between these two isotopes, distinct and complex operational procedures, reaction stoichiometries, and reagent selections are challenges that must be overcome when translating established fluorine-19 methods for fluorine-18 radiochemistry. Through highly productive collaborations between chemists in different (sub)disciplines, modern radiochemical methodologies, including C–H radiolabeling, have emerged to address hurdles in the radiofluorination and radiofluoroalkylation of organic molecules.^7^ In particular, C–H radiolabeling can be conveniently leveraged to overcome precursor stability, prefunctionalization, and toxicity issues that complicate (pre)clinical translation, which frequently manifests in many other traditional and modern radiolabeling precursors.

### 1.1 C–H Radiolabeling with Fluorine-18

Historically, substitution reactions of unactivated C–H bonds have been restricted to harsh chemical processes with low functional group tolerance owing to low kinetic reactivities originating from high C–H bond dissociation energies. Consequently, the hydrocarbon backbone of organic molecules has nominally been considered to have low synthetic utility. However, many ingenious contributions have led to modern and sustainable, metal-promoted/catalyzed^8−12^ and metal-free^13^ C–H activation processes, including organocatalytic,^14^ photochemical,^15^ radical,^16^ electrochemical^17^ reaction systems that can install functional groups efficiently and selectively at (un)activated C–H bonds in densely functionalized organic systems. A complete overview of C–H activation is beyond the scope of this review, although the interested reader is directed to these specialized reviews and articles.

**Scheme 1:**
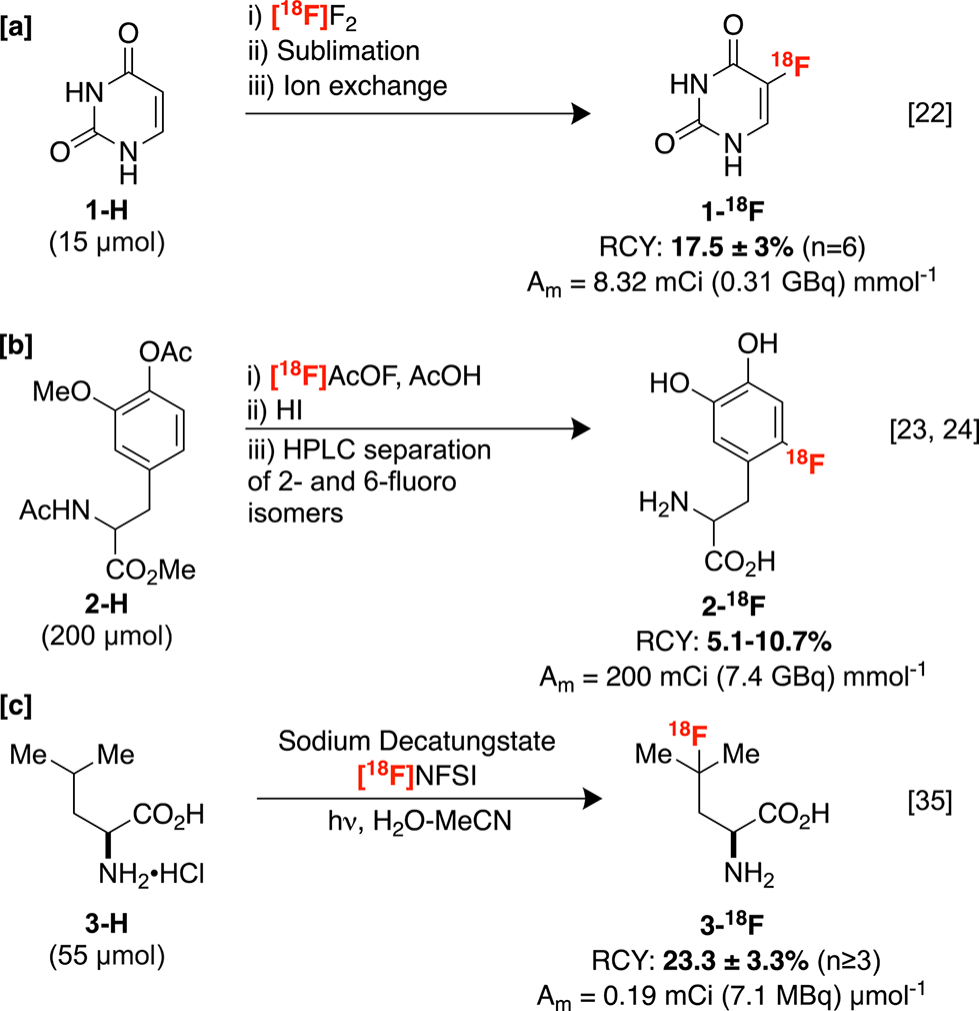
Electrophilic C–H Radiofluorination. 1-18F

Early examples illustrate the challenges of C–H activation in the radiochemical arena, where direct irradiation of organic compounds with nuclear reactor-/synchrocyclotron-produced neutrons was reported to induce C–H substitution with radiofluorine, albeit in poor recoveries and selectivities owing to the harshness of these processes.^18−20^ On the other hand, cyclotron-produced fluorine-18 via the ^16^O(^3^He,p)^18^F or ^20^Ne(d,α)^18^F (for [^18^F]F_2_) nuclear reactions and the ^18^O(p,n)^18^F nuclear reaction (for [^18^F]F_2_ and [^18^F]fluoride) offers improved recovery, higher purity, and is conveniently accessible owing to the now high availability of particle accelerators. Fluorine-18 production in this manner is now routine and available commercially.^21^ A traditional preference for electrophilic fluorine-18 sources such as [^18^F]F_2_ and [^18^F]AcOF has prevailed, with several pioneering studies describing early labeling processes, such as the preparation of archetypal imaging agent [^18^F]fludeoxyglucose (FDG) and electrophilic C–H substitution reactions. For instance, Wolf and co-workers reported the radiosynthesis of a labeled uracil derivative **1-**^**18**^**F** utilized in tumor imaging (Scheme 1A).^22^ Other imaging agents, including 6-[^18^F]F-DOPA **2-**^**18**^**F**, are also prepared with electrophilic fluorine-18 (Scheme 1B), although the regioselectivity of related reactions can be difficult to control, complicating HPLC purification protocols.^23,24^

**Scheme 2:**
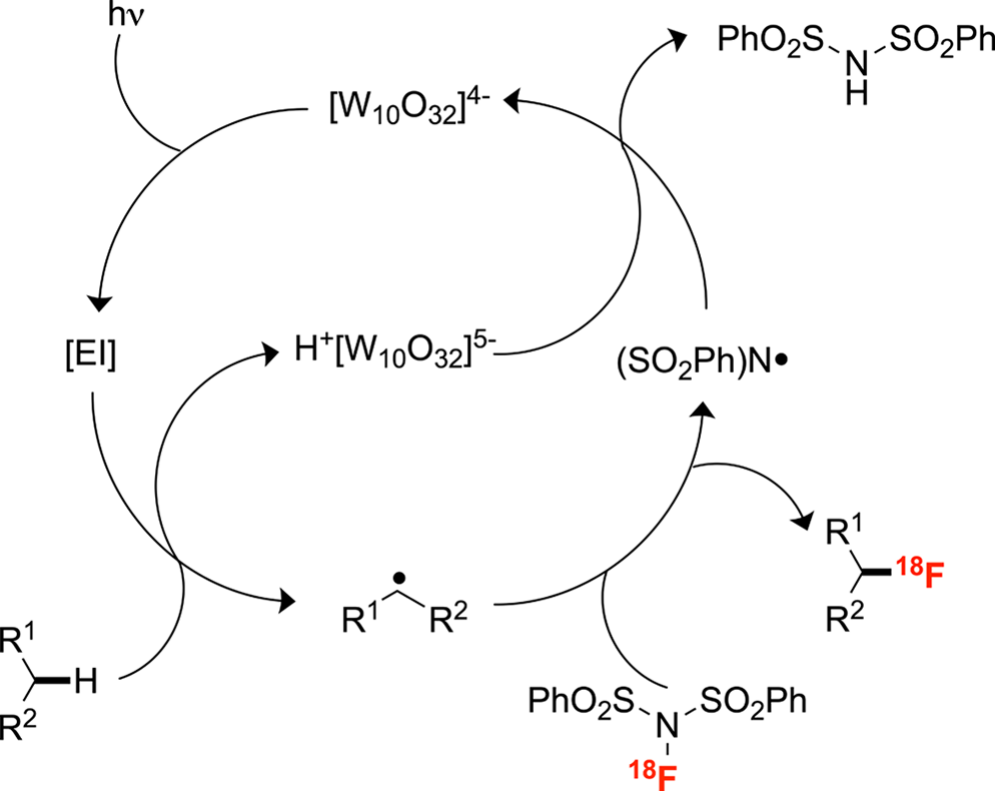
Mechanism of Decatungstate-Mediated Electrophilic C–H Radiofluorination

More recently, N–F reagents,^25−28^ a hypervalent iodine reagent,^29^ [^18^F]XeF_2_,^30^ and Pd complexes^31^ have been developed as alternative non-nucleophilic fluorine-18 sources that can exhibit improved selectivity. Some of these compounds, such as [^18^F]*N*-fluorobenzenesulfonimide ([^18^F]NFSI), may be employed to label C–H bonds of bioactive scaffolds,^32−34^ including branched amino acid **3-H**, which undergoes labeling with selectivity for the tertiary C–H site (Scheme 1C).^35^ This process was inspired by a related nonradioactive C–H fluorination that is proposed to proceed via the photoexcitation of the decatungstate anion, producing a short-lived compound that rapidly decays to an electrophilic intermediate [EI] competent in hydrogen atom abstraction to form the corresponding alkyl radical and H^+^[W_10_O_32_]^5–^. [^18^F]NFSI traps this alkyl radical, furnishing the product and the corresponding *N*-centered radical, which abstracts hydrogen from H^+^[W_10_O_32_]^5–^ to regenerate decatungstate. The corresponding radiofluorination reaction may proceed through an analogous pathway (Scheme 2). Although this radiolabeling method and others discussed in this article can proceed catalytically, stoichiometric pathways that are otherwise impractical in nonradioactive fluorinations become feasible using limiting radiofluoride (*vide infra*).

Despite the utility of electrophilic methods, a shift toward nucleophilic fluorine-18 sources has occurred primarily due to a requirement for nonradioactive ^19^F_2_ carrier gas when generating electrophilic reagents. Molecular fluorine presents operational complexities and limits molar activity (A_m_, the ratio of the desired radioactive agent and nonradioactive carriers) to a maximum of 50%, with much lower values attainable in practice.^36^ Notably, contamination with excess nonradioactive carriers (e.g., fluorine-19 agent analogues) increases competitive target binding, reducing image resolution and presenting toxicity concerns. In contrast, nucleophilic fluorine-18 can be produced with A_m_ values significantly more conducive to imaging, with ca. > 1 Ci μmol^–1^ being optimal for most PET imaging studies. This is possible with small medical cyclotrons, translating to more patient doses from a given radiopharmaceutical batch and images with superior resolution. Furthermore, the reactivity of [^18^F]fluoride is generally easier to control than [^18^F]F_2_ and is more conveniently prepared. Therefore, this article will focus on C–H radiofluorination and radiofluoroalkylation methods that utilize nucleophilic fluorine-18.

In principle, C–H radiolabeling with fluorine-18 shares several advantages with other C–H functionalization reactions, such as (a) a reduced requirement for substrate prefunctionalization, (b) amenability to late-stage functionalization of densely functionalized scaffolds (e.g., bioactive molecules), (c) access to otherwise challenging substitution patterns, and (d) ability to leverage kinetically stable C–H bonds as a synthetic handle.^37^ C–H activation finds additional utility in nuclear medicine as commercial production methods do not require significant scale-up and are, therefore, less restricted by nonradioactive reagent expense. Furthermore, C–H radiolabeling with fluorine-18 offers advantages over other strategies that perturb the original function of a labeled target analogue. For example, routinely conducted prosthetic group strategies typically involve the installation of non-native [^18^F]fluoroalkyl chains that can negatively affect pharmacokinetics, uptake, and binding to targets of interest.^38^ Moreover, C–H radiofluorination and radiofluoroalkylation protocols amenable to late-stage functionalization are highly desirable since radioisotopic decay restricts the number of synthetic steps that can follow radiolabeling. However, for C–H radiolabeling to be effective, the drawbacks of conventional C–H activation should carefully be considered, such as (a) low kinetic reactivity of many C–H bonds, (b) chemoselectivity and C–H site-selectivity control, and (c) the manipulation of directing groups.^39^ Direct nucleophilic C–H fluorination is challenging owing to (a) the typically high basicity and low nucleophilicity of fluoride, (b) the low reactivity of various fluorinated intermediates (e.g., metal fluorides), (c) a high reliance on oxidizing conditions.^40−43^ In addition, fluorine-18 labeling is challenged by the use of (a) substoichiometric [^18^F]fluoride (ca. pmol-nmol quantities), (b) rapid reaction times used to contend with radioactive decay, (c) rigorous controls of dosage purity and release quantities of toxic species (e.g., heavy metals, solvents) from regulatory institutions, such as the International Council for Harmonization of Technical Requirements for Pharmaceuticals for Human Use (ICH), and (d) isotopic ^18^F/^19^F exchange, particularly during elevated temperature reactions where fluorine-19 atoms feature in the substrates/reagents (e.g., radiofluoroalkylation).^44,45^ Critically, C–H radiolabeling procedures should be compatible with commercial radiosynthesis modules to have clinical feasibility due to regulatory considerations in the manufacturing of radiopharmaceuticals (United States Pharmacopeia, USP, 823 and 21 CFR part 212). These modules, which are contained within a lead-shielded chamber (hot cell) to obviate high levels of radiation exposure to the operator, utilize automated radiosynthesis programs to maximize run-to-run reproducibility and record data collected during the manufacturing process for audit. Therefore, C–H labeling protocols should be adaptable to protocols compliant with current good manufacturing practices (cGMP) for clinical adoption.

Typically, it is expedient to develop new labeling processes manually first (i.e., outside of a hot cell with lower levels of radioactivity), although challenges can be faced automating these manual procedures later. Furthermore, automation presents an important consideration when integrating C–H activation methods, especially for producing patient doses. For example, most radiochemical facilities are not equipped to perform more complex transformations (e.g., photoredox or electrochemical reactions), although recently, the feasibility of integrating these technologies has been demonstrated (*vide infra*). Furthermore, the production, handling, and storage of air/moisture-sensitive reagents often used in C–H functionalization are significant challenges for translation to clinical production, as nucleophilic fluorine-18 is produced in an oxygen-18 H_2_O cyclotron target and is typically dried azeotropically.^46,47^ Despite these hurdles, direct and indirect C–H radiofluorination and radiofluoroalkylation reactions are beginning to emerge that can address the drawbacks of state-of-the-art labeling protocols, and this Outlook highlights and discusses recent developments in this field. Molecules labeled using C–H activation are categorized by the hybridization of the carbon center followed by the methodology category.^48^ Unless otherwise stated, nonautomated (i.e., manual) radiochemical yields (RCY) and radiochemical conversions (RCC) refer to nonisolated recoveries of radiolabeled organic products, whereas automated RCYs refer to isolated material.^49^ Although care has been taken to present and discuss these values consistently, this nomenclature is not necessarily uniform across the discussed articles, and the interested reader is invited to clarify terms such as RCC and RCY using the source articles.

## 2 Radiolabeling of sp^2^ C–H Bonds with Fluorine-18

Aromatic sp^2^ C–F bonds are a popular motif in bioactive fluorinated drug molecules and radiofluorinated (pre)clinical PET agents owing to a relatively high metabolic stability. Reflecting this, the following Section highlights how aromatic systems have been a focus of C–H labeling research efforts.

### 2.1 Direct sp^2^ C–H Radiolabeling

#### 2.1.1 Metal-Mediated

**Scheme 3:**
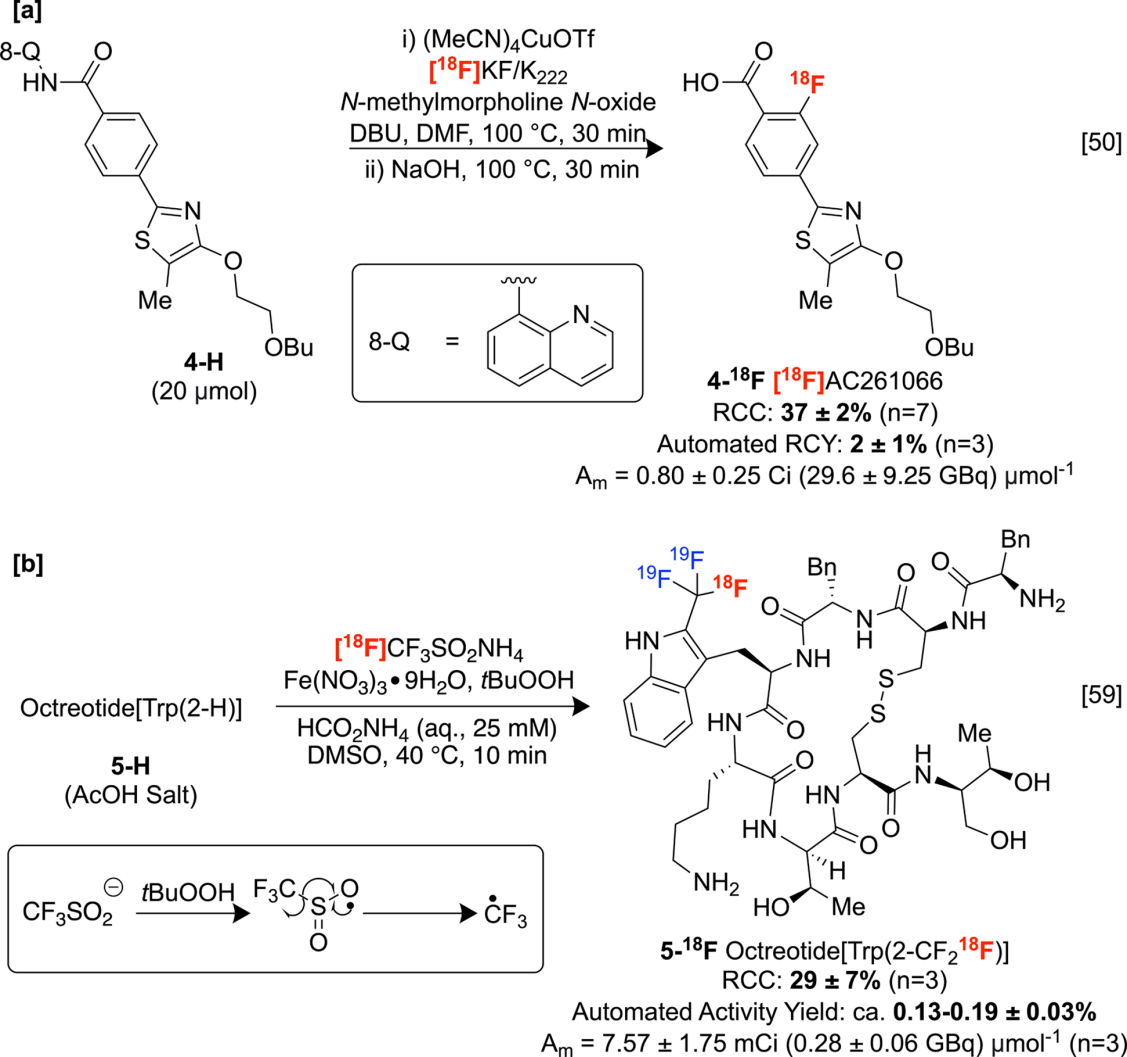
Direct sp^2^ C–H Fluorine-18 Labeling Reactions

Few direct sp^2^ C–H nucleophilic (radio)fluorination and (radio)fluoroalkylation reactions have been communicated, owing to the challenging nature of this transformation. Our laboratories were the first to develop a general, copper-mediated radiofluorination (CMRF) method to access fluorine-18 labeled aromatics directly. This reaction involves an auxiliary-assisted Cu C–H activation of electronically diverse aromatic 8-aminoquinolinyl benzamides (Scheme 3A).^50^ Initial attempts to translate a nonradioactive variant of this reaction previously described by Daugulis and co-workers did not lead to detectable labeling, so several parameters were optimized for application to radiochemistry. For example, switching from [^18^F]AgF to earth-abundant [^18^F]KF significantly improved radiochemical conversions (RCC). Although mechanistic studies of the (radio)fluorination reactions were not disclosed, high *ortho*-site-selectivity is conferred by the aminoquinolyl (AQ) amide moiety, which likely behaves as a bidentate *N*,*N*-ligand to copper in analogy to isolated Pd-8-AQ complexes.^51,52^ This C–H radiofluorination is ideal for translation into clinical production for several reasons. First, carboxylic acids and amides are highly abundant functionalities, comprising >51% of all bioactive molecules in the ChEMBL database.^53^ Second, the derivatization of these motifs to the requisite auxiliary and subsequent deprotection are largely straightforward, so this method may be used to label a vast chemical space of organic molecules conveniently and selectively. Third, copper as a transition metal mediator is ideal owing to a relatively high ICH release limit and permitted daily exposure.^54^ Indeed, densely functionalized substrates were prepared, such as protected RARβ2 agonist [^18^F]AC261066 **4-**^**18**^**F**, successfully automated on a TRACERlab FX_FN_ radiosynthesis module and subsequently obtained as the free carboxylic acid following basic hydrolysis in high radiochemical purity albeit with moderate A_m_.

In analogy to previously reported, nonradioactive C–H trifluoromethylations,^55−58^ Gouverneur and co-workers disclosed a C–H radiotrifluoromethylation of unmodified peptides at tryptophan and tyrosine residues using iron salts (e.g., Fe(NO_3_)_3_•9H_2_O) and *tert*-butyl hydroperoxide.^59^ In this process, peroxide-mediated desulfonylation liberates the trifluoromethyl radical, which is trapped by arenes. This enabled the labeling of complex peptide scaffolds, including an automated [^18^F]trifluoromethylation of somatostatin mimic octreotide **5-H** on an Advion NanoTek microfluidic system (Scheme 3B). Although a radiochemical yield of this process was not disclosed, an activity recovery can be estimated at ca. 0.13–0.19%. Furthermore, the low A_m_ restricts the application of this method for (pre)clinical PET. Since the precursor reagent [^18^F]CF_3_SO_2_NH_4_ is also obtained in low A_m_ (0.01 GBq μmol^–1^), it is possible that carrier fluorine-19 atoms, which feature in the reagents, are introduced during the preparation of this compound. Notably, radical labeling strategies commonly obviate the protection/deprotection of otherwise poorly tolerated functionalities (e.g., OH, NH_2_), streamlining radiosynthetic sequences. However, since the selectivity of these substitutions is related to the relative energies of intermediate organic radicals, this method can face selectivity issues^60^ in other aromatic systems (e.g., *ortho*- vs *para*-selectivity),^61,62^ which also manifests in the electrochemical and photoredox systems (similarly proceeding through radical intermediates) discussed in the following Sections.

#### 2.1.2 Electrochemical

**Scheme 4:**
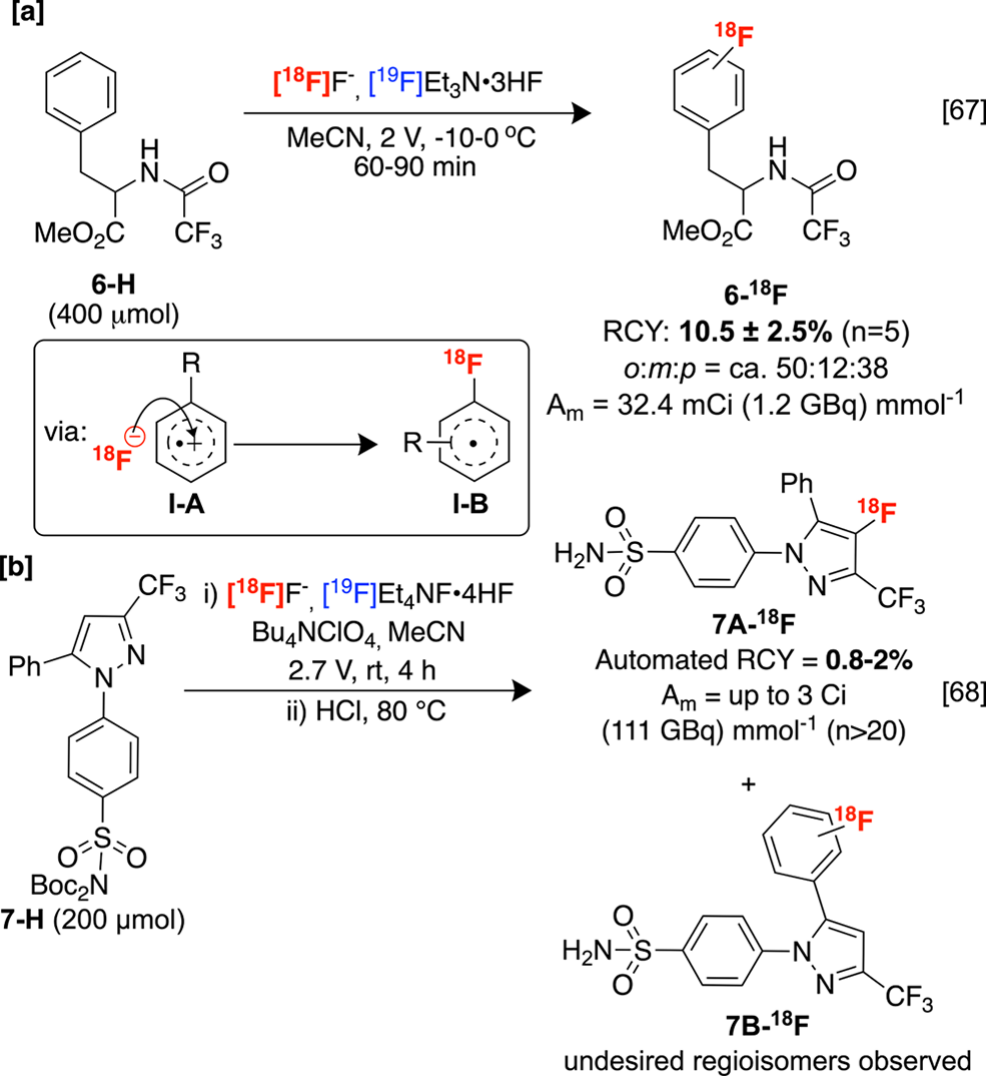
Direct sp^2^ C–H Electrochemical Radiofluorination

Radiolabeling reactions in an electrochemical cell are attractive since they can be used with electrodeposition methods. This involves the separation and release of aqueous fluorine-18 from target [^18^O]H_2_O (from which fluorine-18 is derived) with an electrode, potentially obviating preparatory procedures such as radionuclide elution from a cation-exchange support (e.g., a quaternary methylammonium cartridge, QMA) and azeotropic drying, which diminishes activity yields by prolonging radiosyntheses.^63,64^ Despite this prospect, electrochemical methods are seldom used, although some have been described to promote C–H labeling. For example, Reischl and co-workers described the nucleophilic radiofluorination of aromatic C–H bonds in benzene^65^ and monosubstituted arenes^66^ such as protected phenylalanine derivative **6-H** using electrochemical oxidation with a three-electrode system, which could be conducted on a custom-built automatic radiosynthesis platform. The authors suggest an EC_N_EC_B_ mechanism involving anodic substrate oxidation to radical cation **I-A**, which is trapped by radiofluoride to afford **I–B** under stereoelectronic control. A second oxidation followed by rearomatization affords the labeled products, including regioisomeric product distributions of **6-**^**18**^**F** (Scheme 4A).^67^ This method has limited application in sp^2^ radiolabeling since the obtained A_m_ values do not surpass those commonly obtained using [^18^F]F_2_, which originates primarily from the inclusion of carrier-added ^19^F-Et_3_N•3HF as an electrolyte. The electrochemical radiosynthesis of COX-2 inhibitor **7A-**^**18**^**F** (alongside regioisomeric products **7B–**^**18**^**F**) was later described for monitoring COX-2 expression with rodent PET imaging. This purified imaging agent was obtained with a low A_m_ of up to 3 Ci mmol^–1^, owing to the use of ^19^F-Et_4_N•4HF carrier (Scheme 4B).^68^ As noted by the authors, carrier-added electrolytes are not a fundamental prerequisite to electrochemical radiofluorination, which can be seen in later studies that describe no-carrier-added sp^2^ electrochemical radiofluorodealkylation using Et_3_N•AcOH as a replacement electrolyte. However, this has yet to be employed for the radiofluorination of sp^2^ C–H bonds.^69^ Looking forward, integrating electrochemical radiolabeling methodologies into production facilities hinges on developing labeling strategies that match or surpass the state-of-the-art (particularly A_m_), which could galvanize the widespread installation of electrochemical cells into commercial radiosynthesis modules. Further steps toward this are discussed in Section 3.1.2.

#### 2.1.3 Photochemical

**Scheme 5:**
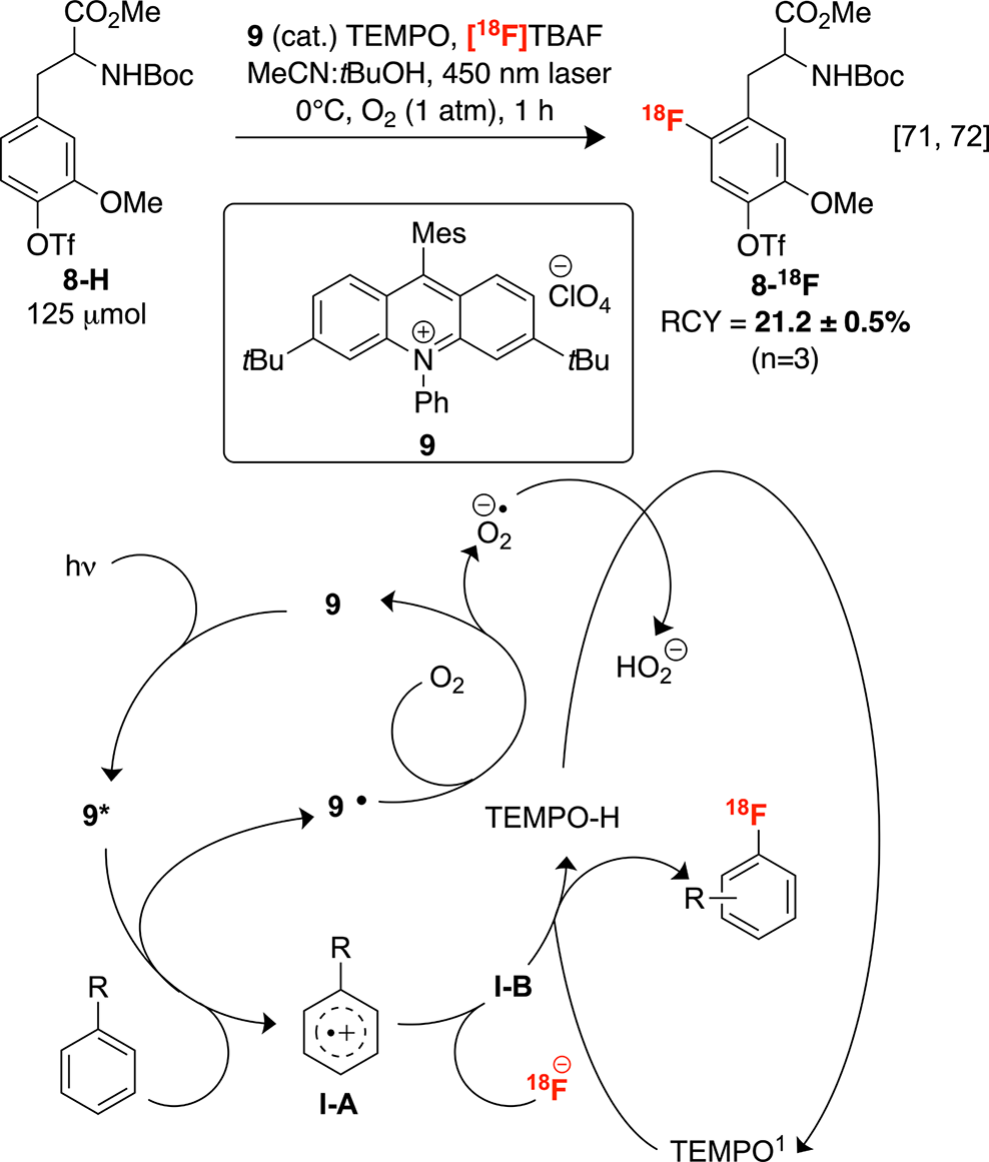
Photochemical C–H Radiofluorination Mediated by **9**, with Plausible Mechanism O_2_ may induce rearomatization.

Photomediated reactions are promising reaction manifolds for producing key reactive intermediates (e.g., aromatic radicals/radical ions)^70^ that ultimately induce or undergo radiofluorination/radiofluoroalkylation, which can reduce or preclude the requirement for high reaction temperatures, harsh redox-active reagents, and fluorine-19 carrier. This could widen fluorine-18 substrate scopes to less thermally stable bioactive scaffolds and mitigate contamination of patient dosages with toxic metal additives. Toward this, Nicewicz, Li, and co-workers employed acridinium salt **9** with a 450 nm laser and TEMPO to generate electron-rich fluorine-18 labeled arenes (Scheme 5). Conceptually, these reactions are mechanistically related to electrochemical C–H radiofluorinations discussed in Section 2.1.2. In contrast, the excited photocatalyst 9* is responsible for arene oxidation and radical cation formation. Trapping of this cation by radiofluoride produces **I–B** (see Section 2.1.2, Scheme 4), which undergoes rearomatization by the action of TEMPO or O_2_ while oxidatively regenerating **9** (Scheme 5). This method was applied to the labeling of scaffolds in low to good RCYs, primarily with *ortho*- and *para*-regioselectivities, such as dopaminergic imaging agent **6-**[**^18^F**]**F-DOPA2-**^**18**^**F**, following deprotection of **8-**^**18**^**F**.^71,72^ Although molar activity for this compound was not disclosed, fluorine-18 labeled 4-phenoxybenzene was obtained under these conditions in high A_m_ at 1.37 Ci μmol^–1^. Although this is applicable to a range of bioactive scaffolds, an automated protocol has yet to be described.

**Scheme 6:**
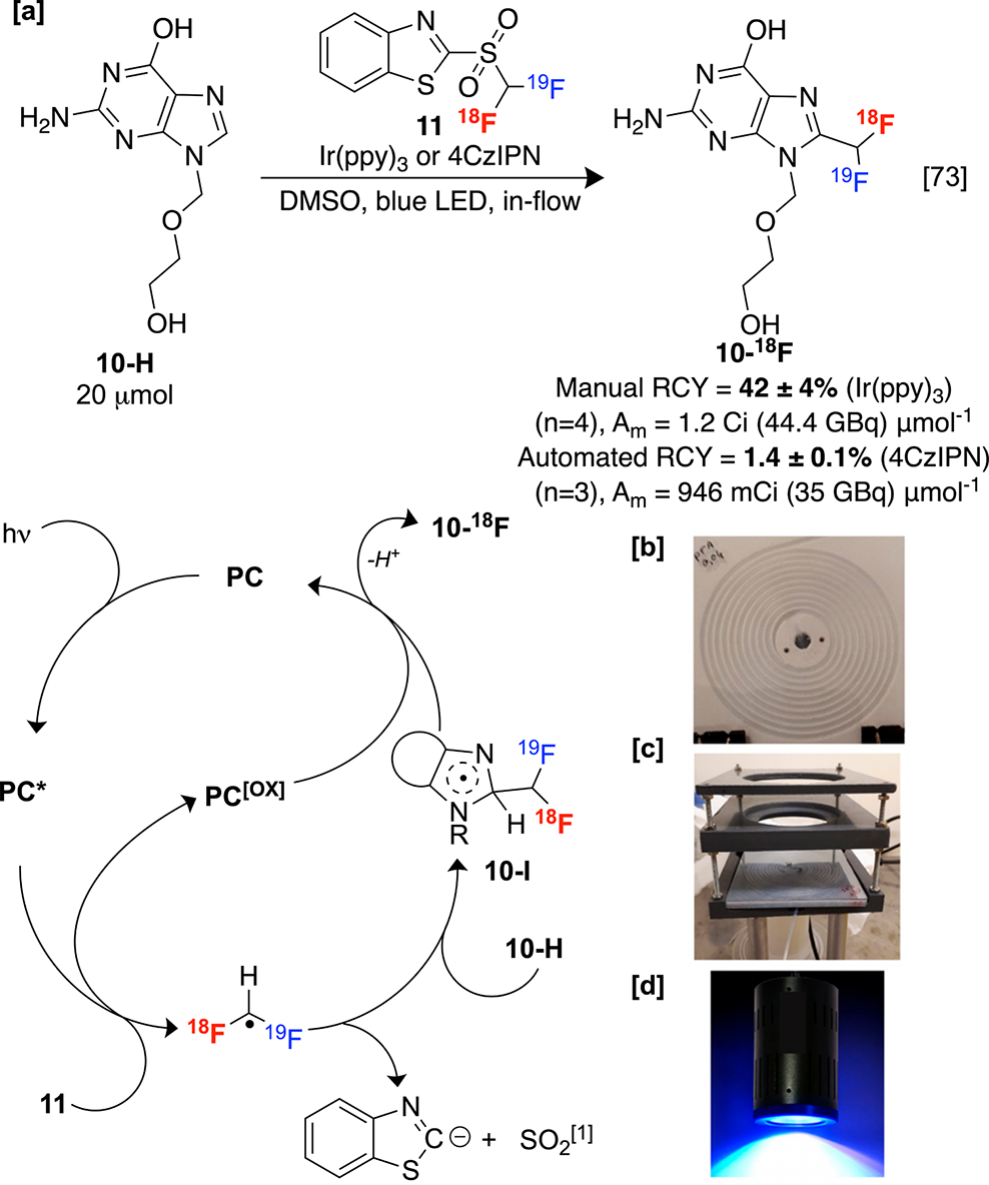
Photochemical C–H Radiofluoroalkylation Starting from **11**, with Plausible Mechanism Sulfinate byproduct may be produced.

Luxen, Genicot, and co-workers disclosed a [^18^F]difluoromethylation of nitrogen-rich heterocycles in the presence of photocatalysts (e.g., tris(2-phenylpyridine)iridium, Ir(ppy)_3_ or 1,2,3,5-tetrakis(carbazol-9-yl)-4,6-dicyanobenzene, 4CzIPN) by exposing prelabeled **11** to blue LED irradiation and liberating an active difluoromethylating species, likely the [^18^F]difluoromethyl radical.^73^ This radical is generated via reductive fragmentation induced by an excited-state photocatalyst (PC*), which is recycled by dearomatized compounds produced upon the addition of [^18^F]difluoromethyl radical into arenes (e.g., **10-I**, Scheme 6). Although C–H labeling is remarkably fast (2 min), the relatively cumbersome two-step process required generating **11** limits yields owing to radioactive decay. Like other radical labeling processes, this reaction tolerates commonly problematic functionalities, including protic groups that may otherwise require protecting group strategies. For instance, antiherpetic acyclovir **10-H** was labeled in high RCY and A_m_ conducive to *in vivo* PET studies. However, modest site selectivity was recorded in some precursors containing multiple C–H bonds.

Later, Genicot, Luxen, and Lemaire disclosed a fully automated and stepwise production of **10-**^**18**^**F** in flow with an organic photocatalyst on a Trasis AllinOne (AIO) synthesis module with A_m_ values comparable to the seminal report, significantly improving prospects for this method to be adopted clinically. The authors designed and integrated a spiral-printed reactor with perfluoroalkoxyalkane tubing (Scheme 6b) and apparatus support (6c) to accommodate the blue Kessil LED light (6d). More recently, other groups have also described the assembly of apparatus required for automated photochemical radiosynthesis.^74^ Notably, labeled difluoromethyl groups seldom feature in imaging agents, reflecting the difficulty of this transformation and the propensity of many protocols to induce deleterious isotopic dilution.

### 2.2 Indirect sp^2^ C–H Radiofluorination

Two-step, one-pot methodologies are attractive alternatives to direct C–H radiolabeling since they do not necessarily require the development of mechanistically novel processes and can instead draw upon the wealth of available C–H activation and C–X radiolabeling protocols. Typically, this approach incorporates activation-labeling steps to furnish fluorine-18 containing aromatics, which, in analogy to direct C–H radiofluorination, can circumvent the isolation, handling, and long-term storage of independently generated, unstable intermediates.

#### 2.2.1 Sequential Protocols

**Scheme 7:**
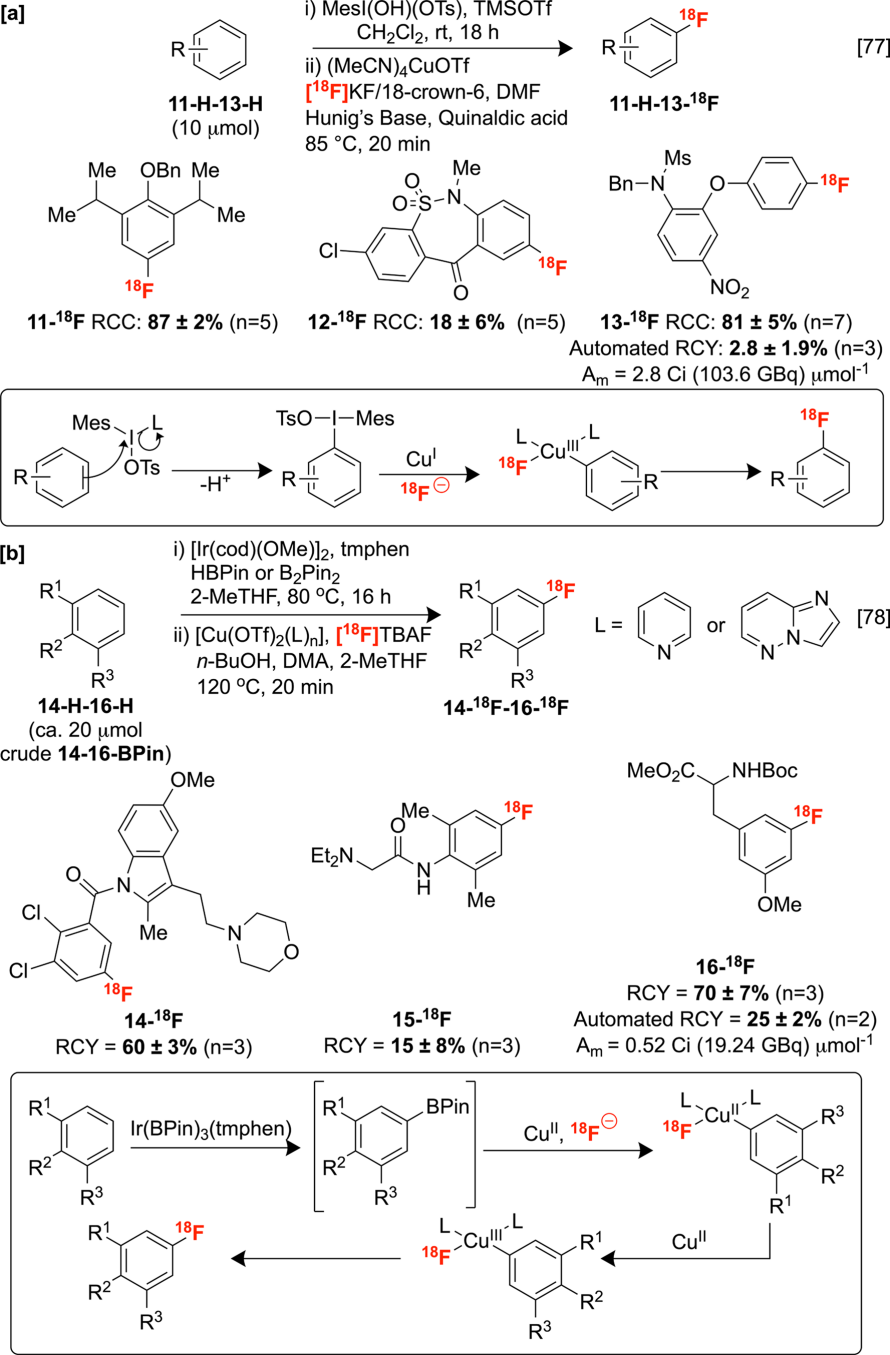
Sequential C–H Radiofluorination Protocols

Our groups have contributed clinically applicable approaches for labeling aromatic C–H bonds by combining widely adopted CMRF methodologies^75,76^ with operationally convenient and robust C–H activation processes. For example, we reported the tandem radiofluorination of electron-rich (hetero)arenes by first incorporating an activation step of (hydroxytosyloxyiodo)mesitylene with TMSOTf to induce S_E_Ar C–H substitution.^77^ Notably, the high steric bulk of the hypervalent I^III^ intermediate generated *in situ* facilitates high *para*-regioselectivity, which is generally challenging to induce with C–H functionalization. Following activation, the (mesityl)(aryl)iodonium intermediate undergoes oxidative addition to copper with high chemoselectivity for the least sterically congested aryl substituent. Reductive elimination from the resulting aryl Cu(III) radiofluoride furnishes the new C–^18^F bond. The applicability of this approach to electron-rich arenes addresses issues with traditional radiofluorinations that generally require electron-deficient substrates (e.g., S_N_Ar). Notably, this method carries promise for clinical translation, as exemplified by the automated radiosynthesis of NSAID derivative nimesulide **13-**^**18**^**F** on a GE TRACERlab FX_FN_ module (Scheme 7A).

Our groups later reported a sequential fluorine-18 labeling of (hetero)arenes by first incorporating iridium-catalyzed C–H borylation to deliver the corresponding aryl pinacolatoboryl esters (BPin) with sterically controlled regioselectivity. Following C–H activation, the intermediate organoborons undergo CMRF promoted by *n*-BuOH, which is believed to sequester residual HBPin and enhance the conversion of the labeling step. The *meta*-regioselectivity conferred by the crowded [Ir(BPin)_3_tmphen] active complex renders this method complementary to tandem labeling via iodonium intermediates. Transmetalation of the *in situ* generated aryl boronate to Cu^II^, followed by disproportionation and reductive elimination, facilitates a net C–H to C–^18^F transformation. By judicious selection of [Cu] complexes reported by Gouverneur, the labeling of both electron-rich (L = pyridine, py) and electron-deficient (L = imidazo-[1,2-*b*]pyridazine, impy) aromatics was accomplished (Scheme 7B), including an automated radiosynthesis of protected tyrosine derivative **16-**^**18**^**F** in RCYs suitable for clinical production.^78^

#### 2.2.2 Multistep Approaches

Other indirect C–H radiofluorination protocols that require the isolation of activated intermediates have also been described. For example, Gouverneur and co-workers reported the preparation of copper azacalix[1]arene[3]pyridine complex **18** from the corresponding C–H precursor **17**(79,80) (Scheme 6A).^81^ The critical C–H activation process that affords the well-defined Cu^III^ species **18**, capable of undergoing reductive elimination, likely proceeds by one of two distinct pathways. The first involves electrophilic metalation to an aryl Cu(II), which is then oxidized by another Cu(II) ion. The second is a proton-coupled electron transfer mechanism, in which a nonconventional interaction between Cu(II) and the key aromatic C–H bond facilitates coupled deprotonation and oxidation by another Cu(II) ligand complex.^82^ Although this radiochemistry is not currently applicable in the clinic owing to the use of carrier-added ^19^F-TBAF•(pin)_2_, in-depth studies have been disclosed concerning the Cu C–H functionalization of related macrocyclic substrates, which could permit this method to be applied to a broader scope of organic molecules of clinical relevance.^83^

**Scheme 8:**
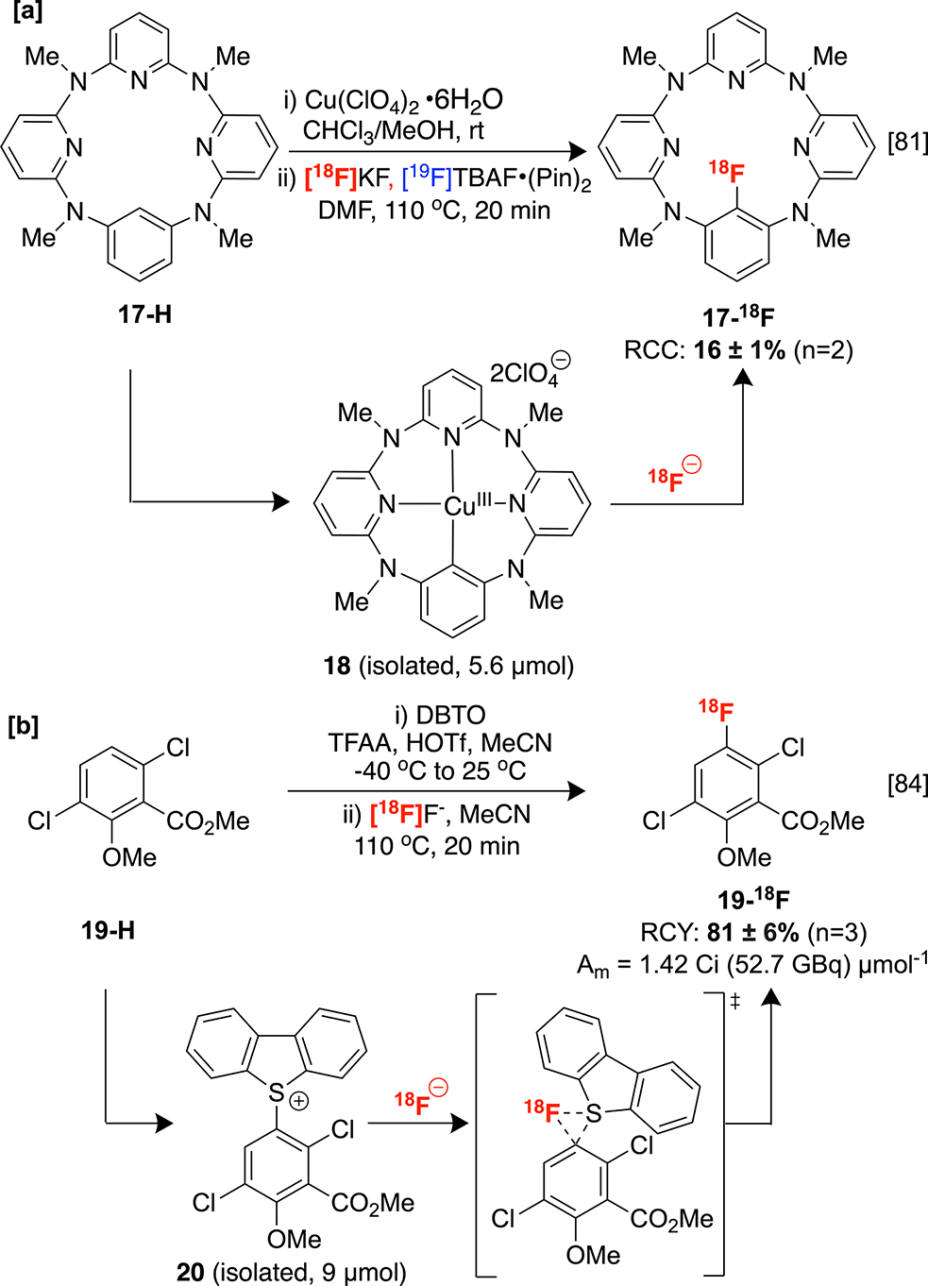
Multistep sp^2^ C–H Radiofluorination Methodologies

Ritter and co-workers disclosed a two-step C–H radio fluorination of (hetero)arenes by generating dibenzothiophenium triflate salts such as **20** via electrophilic aromatic substitution with *S*-oxide derivatives (e.g., dibenzothiophene *S*-oxide, DBTO) with high *para*-selectivity in moderate to excellent RCYs.^84,85^ Notably, these substrates could be used to directly elute fluorine-18 from a QMA cartridge. Upon heating with nucleophilic fluorine-18, these salts readily undergo *ipso*-radiofluorination in analogy to other triaryl sulfoniums reported previously.^86^ The labeling of multiple bioactive scaffolds was achieved according to this protocol, including an automated radiolabeling of protected herbicide dicamba **19-H** on an ELIXYS FLEX/CHEM radiosynthesis module (Scheme 8B). The radiofluorination step of this protocol is attractive owing to its relatively high operational simplicity and metal-free conditions. However, obtaining the requisite dibenzothiophenium salts derived from the strongly acidic and oxidizing C–H functionalization step is an essential consideration that may limit broad clinical translation.

## 3 Fluorine-18 Radiolabeling of sp^3^ C–H Bonds

Fluoroalkyl motifs are another useful handle in bioactive molecules for radiochemists to exploit in fluorine-18 labeling.^87^ Aliphatic C–F linkages occur in many pharmaceuticals, which can impart beneficial properties, such as increased metabolic stability and lipophilicity.^88^ With the current focus on nucleophilic fluorine-18, classical methods of sp^3^ radiofluorination generally consist of S_N_2 reactions of prefunctionalized substrates (e.g., halides, sulfonates),^89^ which can be time-intensive to prepare and can suffer from limited stability and storability. In addition, specific linkages, such as tertiary C–F bonds, are not generally accessible through classical radiofluorination methods due to competing elimination reactions, although strategies involving labeling activated intermediates have enabled this transformation.^90^ In contrast, C–H radiofluorination could facilitate rapid labeling of a library of precursors selectively at 1°, 2°, or 3 °C–H bonds. Despite the high bond-dissociation energy of sp^3^ C–F bonds (H_3_C–F = 107 kcal mol^–1^),^91^ it is important to note a common kinetic susceptibility to metabolism *in vivo*, (e.g., via dehydrodefluorination or glutathione nucleophilic displacement) particularly in activated, uncongested sp^3^ systems such as primary benzylic fluorides. This decomposition can lead to poor imaging resolution due to the accumulation of [^18^F]fluoride in nontargeted areas, such as bone.^92^ Nevertheless, numerous imaging studies feature sp^3^ C–^18^F labeled compounds, with [^18^F]FDG as an important example.^93^ Furthermore, emerging sp^3^ C–H radiofluorination strategies have incorporated strategies to mitigate metabolic defluorination. To date, there are limited reports of nucleophilic sp^3^ C–H [^18^F]fluorination methods, which are highlighted in the following Section.

### 3.1 Direct sp^3^ C–H Radiolabeling with Fluorine-18

#### 3.1.1 Metal-Mediated

**Scheme 9:**
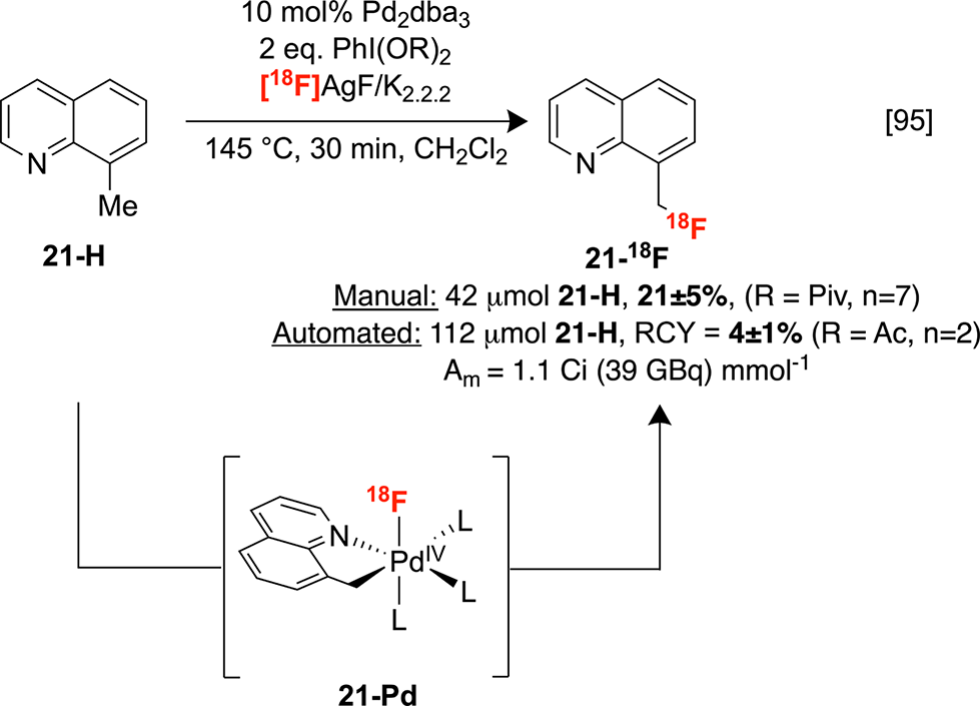
Directed Radiofluorination of sp^3^ C–H Bonds

The first example of nucleophilic sp^3^ C–H ^19^F-fluorination via cross-coupling was developed by Sanford for the benzylic fluorination of 8-methylquinoline derivatives.^94^ The combination of AgF as a nucleophilic fluoride source and PhI(OPiv)_2_ as an oxidant facilitates C–H fluorination through a proposed cyclometalated Pd^IV^ intermediate, with the quinolinyl fragment behaving as a directing group (DG). Our laboratories successfully translated this system with modifications, which may feature related fluorine-18 intermediates such as **21-Pd**.^95^ No radiofluorination was observed when [^18^F]KF was used as the fluoride source, prompting the development of a general procedure for preparing high molar activity [^18^F]AgF by eluting radiofluoride with different Ag salts. In analogy to the nonradioactive reaction, [^18^F]AgF provided good yields across several 8-methylquinolines bearing a range of groups at the 5-position, including **21-H**. Furthermore, this method was amenable to automated radiosynthesis using a GE TRACERlab FX_FN_ synthesis module, affording **21-**^**18**^**F** in 4 ± 1% isolated RCY and modest molar activity (Scheme 9). The obtained RCYs, substrate scope, and use of hazardous metals need to be accounted for during future translation of this method into a clinical setting. Nevertheless, this study represents an essential proof-of-concept for nucleophilic, directed sp^3^ radiofluorination that could serve as a blueprint for the future design of related systems with broader applicability.

**Scheme 10:**
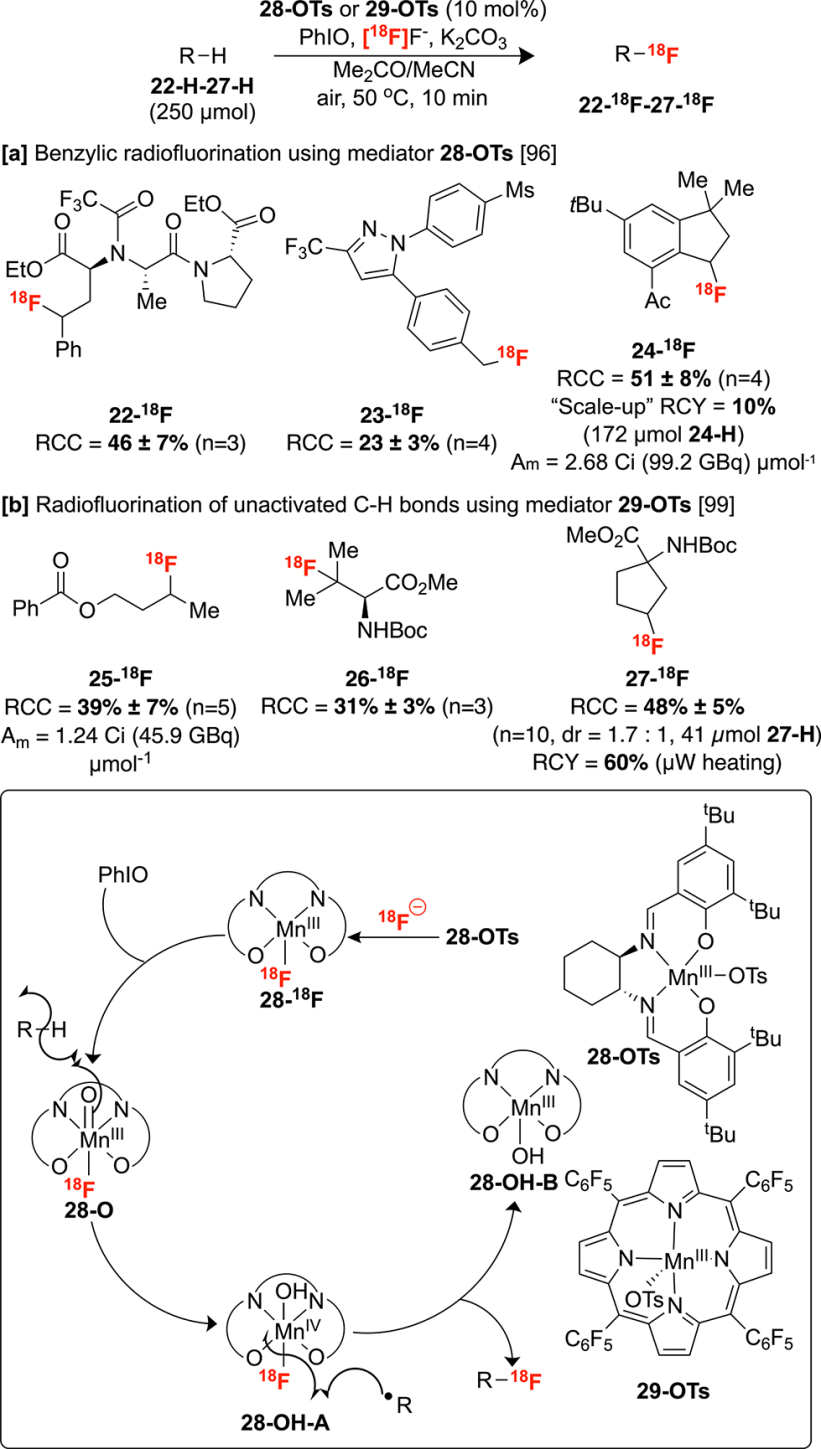
Manganese-Mediated Radiofluorination of sp^3^ C–H Bonds

Protocols that enable C–H radiofluorination without a DG are desirable because they are applicable to a broader range of substrates and may obviate post-radiolabeling steps. Groves and Hooker demonstrated undirected benzylic C–H radiofluorination using radiofluoride in conjunction with PhIO and bulky Mn(salen)OTs complex **28-OTs**.^96^ This protocol was translated from a ^19^F fluorination reported previously by the same group. Intriguingly, the radiofluorination exhibits a broad substrate scope, tolerating β-electron withdrawing groups such as Boc-amines and ketones not amenable in the ^19^F method. This was proposed to be a result of the low concentration of [^18^F]fluoride with respect to the Mn precatalyst. Additionally, several bioactive molecules undergo benzylic radiofluorination in moderate to good RCCs, including protected ACE inhibitor enalaprilat **22-H**, COX-2 selective inhibitor celecoxib analogue **23-H** and celestolide **24-H** (Scheme 10A). A Mn(IV) carbonyl complex **28-O** formed upon the action of PhIO on **28-**^**18**^**F** was suggested to initiate C–H bond homolysis, forming the corresponding alkyl radical, which abstracts radiofluoride from **28-OH-A**. The participation of the Mn(salen) complex in the C–^18^F bond-forming step was demonstrated through chiral HPLC analysis of the products: (R,R)-**28-OTs** gives 25% ee of **24-**^**18**^**F**, while (L,L)-**28-OTs** gives 25% enantiomeric excess but with reversed enantioselectivity. An advantage of this system is its practicality since the reaction can be performed under air without rigorous exclusion of water, and an organic solution of the Mn precatalyst can be used to directly elute ^18^F^–^ during fluoride preparation without an azeotropic dry-down procedure. There after, Carroll and co-workers extended this radiosynthesis to other systems, including [^18^F]trifluoromethylated arenes using the chloride analogue of **28-OTs** under analogous conditions.^97,98^

Groves and Hooker subsequently disclosed a Mn-mediated radiofluorination of unactivated sp^3^ C–H bonds under a mechanistically related system using a combination of ^18^F^–^, PhIO, and K_2_CO_3_.^99^ While only trace amounts of aliphatic labeled products were observed with Mn(salen)OTs **28-OTs**, switching to the bulky, electron-deficient porphyrin tetrakis(pentafluorophenyl)porphyrin (TPFPP) ligand in **29-OTs** led to improved reactivity. Moderate to good yields were obtained for several substrates, displaying selectivity for the most sterically accessible and electron-rich methylene or methine moieties (Scheme 10B). Several bioactive molecules, including amino acids **25–27-H**, were labeled with excellent selectivity for tertiary/secondary C–H sites, furnishing tracers that are more challenging to access through standard nucleophilic substitution. Scaling the labeling reaction of **27-H** to produce clinically useful doses required microwave heating, which may present challenges to translation since cGMP radiosynthesis modules are not currently commercially available to perform such reactions.

**Scheme 11:**
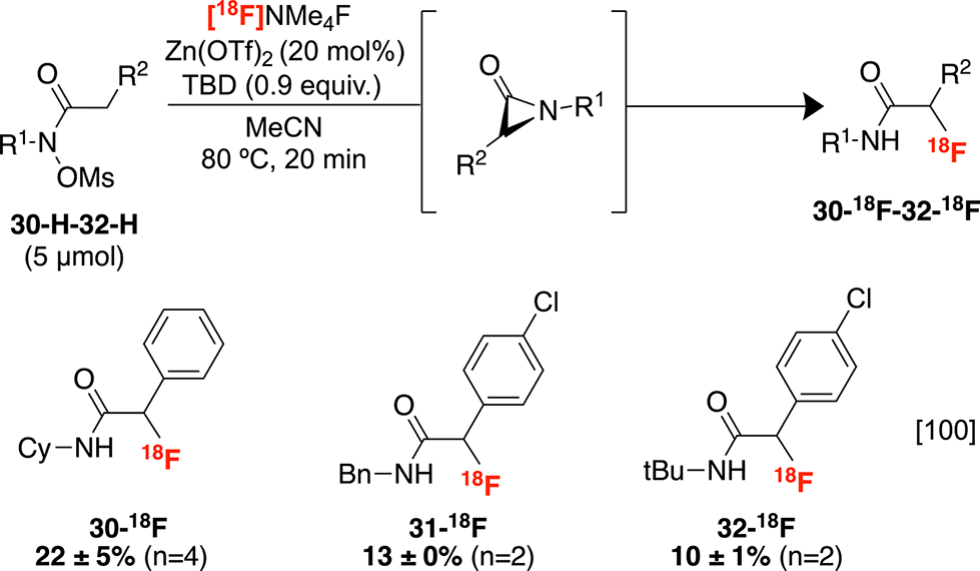
Directed Radiofluorination of sp^3^ C–H Bonds

Recently, our laboratories described a highly efficient radiofluorination of sterically congested α-haloamides.^100^ It was subsequently shown that these reaction conditions were also amenable to the radiolabeling of *N*-sulfonyloxyamides, constituting a formal nucleophilic C–H radiofluorination via an umpolung labeling strategy. Specifically, the C–H precursors **30-H-32-H** generate highly strained α-lactam intermediates on treatment with triazabicyclodecene (TBD), which can rapidly trap radiofluoride to afford the corresponding unprotected α-2°-radiofluoroamides (Scheme 11). Although the conversions of this transformation are modest, they are sufficient for preclinical applications. Our laboratories are currently developing reaction conditions tailored to the efficient and automated C–H radiofluorination of these substrates.

#### 3.1.2 Electrochemical

**Scheme 12:**
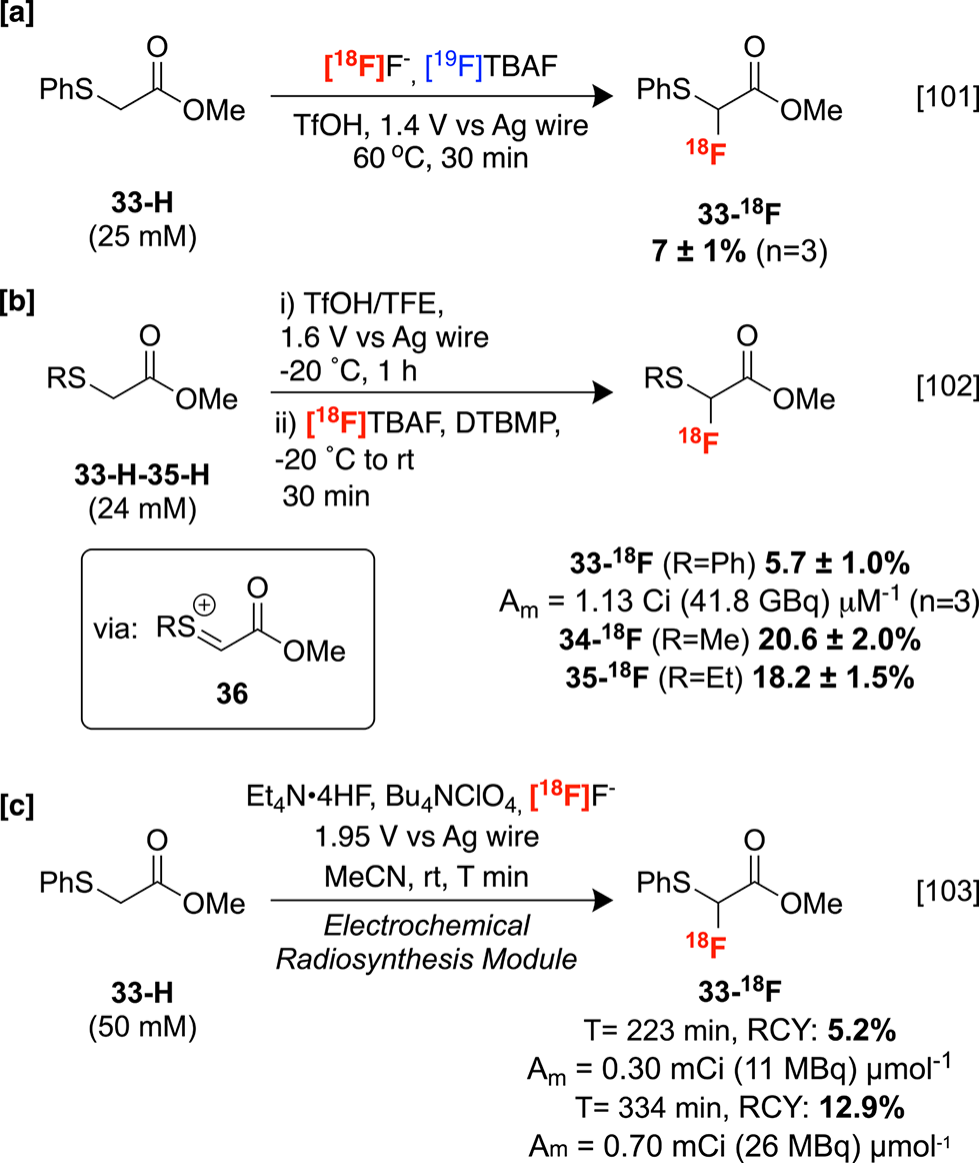
Electrochemical sp^3^ C–H Radiofluorination

Electrochemical radiofluorination of sp^3^ C–H bonds has been explored, although the substrate scope remains limited to a handful of substrates with activated C–H bonds. These strategies face many of the challenges of sp^2^ electrochemical radiofluorination discussed in Section 2.1.2, particularly low A_m_ values. By way of example, Sadeghi and co-workers have demonstrated carrier-added radiofluorination of methyl(phenylthio)acetate **33-H** under controlled potentiostatic conditions in 7 ± 1% RCY (Scheme 12A).^101^ Subsequently, the same group applied a stepwise “cation pool” approach, consisting of initial anodic two-electron oxidation at low temperatures to give cationic intermediates such as **36**, which are trapped by [^18^F]TBAF in the presence of 2,6-di*tert*-butyl-4-methylpyridine (DTBMP) to furnish multiple α-labeled derivatives **33-**^**18**^**F-35-**^**18**^**F** without fluorine-19 carrier while preventing overoxidation (Scheme 12B).^102^ Furthermore, the authors have designed a preliminary electrochemical synthesizer system for the automated, carrier-added production of **33-**^**18**^**F** in good RCYs, albeit with relatively long reaction times (Scheme 12C).^103^ Although electrochemical fluorine-18 C–H radiolabeling remains relatively underdeveloped, it is evident from these studies that some shortcomings are beginning to be independently addressed, raising promise for adopting this technology in a clinical setting. Ultimately, combining these innovations to produce high A_m_ fluorine-18 imaging agents electrochemically would be desirable. Ideally, such a system would operate under automated conditions while minimizing reagents that are typically incompatible with components of commercial radiosynthesis modules (e.g., very strong Brønsted acids).

In this Outlook, C–H radiolabeling techniques with nucleophilic fluorine-18 have been showcased and briefly evaluated based on prospective clinical applicability. These transformations enable access to fluorine-18 PET agents that are otherwise challenging to construct using traditional radiochemical methodologies. However, C–H labeling with nucleophilic fluorine-18 remains in its infancy within the radiochemical arena, with no PET imaging agents currently produced clinically with these approaches. Of the innovations discussed, copper-mediated C–H labeling approaches offer some of the highest radiochemical yields and molar activities while minimizing precursor quantities and handling activated intermediates. Additionally, these methods have automated production procedures ready for clinical adoption. Besides cost reduction, precursor quantity is an often-overlooked parameter in radiochemistry since standard semipreparative HPLC purifications have diminished resolution when overloaded with organic reaction components, complicating isolations, and reducing chemical purity in some instances.

Replacing established labeling methods is a logistically time-consuming endeavor owing to extensive regulatory procedures that ensure a new protocol’s reproducibility and safety in the routine production of patient doses for clinical imaging. To justify this, the production of fluorine-18 imaging agents with new C–H radiolabeling protocols should aim to meet several criteria, such as comparable or enhanced RCY and A_m_ over established processes, simple quality control procedures, and access to targets with optimal PET imaging properties while remaining compliant with guidelines from regulatory institutions, such as the ICH, USP, and FDA. This final point is particularly relevant to methodologies that utilize toxic materials (e.g., toxic metals, solvents), which can complicate quality assurance analyses and potentially contaminate patient dosages. This hinges on the translation and development of highly efficient C–H radiosynthetic protocols, although the lack of existing nucleophilic ^19^F systems presents an obstacle to this goal. In contrast, numerous electrophilic systems support a wide scope of C–H ^19^F fluorinations. Adapting these methods by instead utilizing a suitable combination of [^18^F]fluoride and a replacement oxidant for [F^+^] is one strategy in use that may facilitate the innovation of nucleophilic methodologies that are translatable to PET clinical production. As radiofluorination reactions need to be rapid (ca. < 1 h) to contend with radioactive decay, transient generation of highly electrophilic intermediates, such as carbenes, high valent metal fluoride, cations, and strained ring systems that can rapidly be trapped by radiofluoride may also facilitate the development of new C–H radiofluorination reactions.

## 4 Future Directions

In summary, C–H radiolabeling presents a promising approach for expanding available radiochemical space in the radiofluorination and radiofluoroalkylation of small organic molecules for PET imaging. Furthermore, late-stage C–H labeling with fluorine-18 is an attractive approach for supporting rapid drug-target engagement screening to guide the development of new therapeutics. Conveniently, C–H radiolabeling frequently circumvents the isolation/handling of more procedurally problematic substrates (e.g., classes of organoborons, iodoniums, sulfoniums, and sulfonates), which necessitate cumbersome prefunctionalization procedures and carry limited storage capacities.^104^ Given the numerous successes of C–H activation in various settings (e.g., academic, industrial), we expect that the current landscape discussed in this Outlook, in conjunction with future developments, will render C–H radiolabeling as an invaluable tool for the radiosynthesis of established and novel fluorine-18 agents used in proof-of-concept and (pre)clinical PET imaging studies. Building on the multidisciplinary nature of nuclear medicine, further collaborative efforts between physical and imaging scientists with healthcare providers would help orient new radiochemical research directions, ultimately expediting access to new radiopharmaceuticals and improving patient outcomes.

## Supporting Information Available

Transparent Peer Review report available (PDF)
