# Supplementary material for: C–H Labeling with [18F]Fluoride: An Emerging Methodology in Radiochemistry
Source: ACS Cent Sci. 2024 Aug 23;10(9):1674–88. doi: 10.1021/acscentsci.4c00997 (PMC11447958; doi:10.1021/acscentsci.4c00997)
Supplement: Supplementary file 1 — oc4c00997_si_001.pdf [file oc4c00997_si_001.pdf]

oc-2024-009976.R1

Name: Peer Review Information for "C–H Labeling with [¹⁸F]Fluoride: An Emerging Methodology in Radiochemistry"

First Round of Reviewer Comments

Reviewer: 1

Comments to the Author
The review article summarizes radiochemistry for C-H radiolabelling methods with [18F]fluoride. The review addresses a highly innovative topic in current 18F radiochemistry to narrow critical gaps between 19F and 18F chemistry advancements.
The review is prepared by leaders in the field, providing an up-to-date and comprehensive overview of C-H labelling strategies and methods with readily available [18F]fluoride. The review is very well-organized, reflecting on the opportunities and challenges of C-H radiolabeling procedures from a radiochemist perspective, clearly focusing on technology dissemination toward clinical translation and clinical application.
The authors illustrate different C-H functionalization chemistry with 18F by providing several examples explaining the discussed chemistry. The provided schemes are critical to following the discussion in the text. A better alignment of the schemes with the text would help to improve readability (E.g. direct sp2 C-H radiolabeling is discussed on page 5, but the corresponding schemes are shown on page 3). Moreover, all schemes should use the same font size and size of presented compounds for better readability.
The review discusses highly innovative but also complex chemistry using metal and metal-free C-H activation processes. If known, the authors should provide additional information, including schemes on the mechanisms of the discussed C-H radiolabelling method. This would make the review an excellent source of information on the current state-of-the-art C-H labelling strategies with [18F]fluoride and a valuable educational experience on complex reaction mechanisms in the domain of 18F radiochemistry.
Overall, this is an excellent review addressing an important research topic. The review is timely and very well written. The research community will highly appreciate the summary of C-H labelling methods with [18F]fluoride.

Reviewer: 2

Comments to the Author
This ‘Outlook’ describes the current state of C-H radiofluorination methods and details the challenges of these methods in terms of selectivity, precursor stability (and prefunctionalization needs), yield, molar activity, late-stage functionalization, access to densely functionalized substrates, etc. Overall, the topic is well researched and reported with appropriate references and a thorough report on the various C-H radiofluorination methodologies. A few typos and minor suggestions to improve the manuscript are listed below:

- The authors are inconsistent with their description of molar activity. In the field, an optimal or high molar activity is considered > 1 Ci per umol. The authors state this parameter in the second page of their manuscript as well. However, in section 2.1 when describing the compound

labeled 4-^{18}F, the authors state it is obtained in high radiochemical yield and Am (the reported Am in Scheme 2a is 0.80 Ci per umol, which is moderate). Again, in the following paragraph, the authors describe compound 5-H and state that radiofluorination of 5-H was automated on a microfluidic system in high Am (the reported Am in Scheme 2b is 7.57 mCi per umol, which is very low). Again, in section 2.1.3, in the first paragraph the authors state "4-phenoxybenzene was obtained in 1.37 Ci per umol Am, sufficient for most preclinical studies". This should be considered high Am, the use of the word "sufficient" seems misplaced. Again, in section 2.1.3, in the second paragraph, the authors describe 10-H and state it was "labeled in high RCY and Am" (the Am reported in Scheme 4B is 1.2 Ci per umol and 0.946 Ci per umol, this is lower than the Am obtained for 4-phenoxybenzene for which the authors called "sufficient"). These inconsistencies should be corrected.

- In Scheme 3b, there are multiple compounds shown and one yield reported. Is the automated yield reported for the purified 7A-18F (the authors state in the manuscript that automated recoveries refer to isolated material)? As depicted, it looks like there is an inseparable mixture of compounds. Consider revising the figure to clarify the yield is for the purified 7A-18F.
- In section 3.1.1, the authors describe Scheme 7 in the first paragraph, but it is not referred to in the text. Same is true for the second paragraph of section 3.1.1 and Scheme 8. Please add "Scheme 7" and "Scheme 8" in the appropriate text, when referring to those schemes. In the last paragraph of section 3.1.1, describing compounds 30-32-H, the authors refer to Scheme 8. This is a typo – should be Scheme 9.
- For Scheme 2A, I believe the labeling of compound [18F]AC261066 should be written "18F-AC261066" as the brackets are only used when a fluorine atom is being described or is present the name of the compound (which is not the case here).
  Refer to nomenclature rules detailed in this article:

  https://www.sciencedirect.com/science/article/pii/S0969805117303189?via%3Dih ub
- In Scheme 2B, it would be better to show the structure of the product, with the $CF_2{}^{18}F$ group in the product, in red. The readers can deduce (given the topic of the article and the structure sequences in all the schemes) that the reactant has an 'H' where the '$CF_2{}^{18}F$' group is located. I think this would be clearer than the current state of Scheme 2B.
- Lastly, the authors should be careful with the tone of the article when describing their own methodologies versus other groups' reported methodologies. The Sanford and Scott groups have developed numerous, highly valuable methods and many are described in this manuscript. In an Outlook such as this, it is critical to describe each method with a non-bias eye. I would suggest the authors review their manuscript with this mind. For example, in the first paragraph of section 2.2.1, the authors describe their own methodology and state . . . "facilitates excellent pararegioselectivity", "with excellent chemoselectivity", "carries excellent prospects". In one paragraph, the word 'excellent' is used three times. . . consider revising.

Author's Response to Peer Review Comments:

Response to Reviewers

Reviewer 1

- *"The review article summarizes radiochemistry for C-H radiolabelling methods with [18F]fluoride. The review addresses a highly innovative topic in current 18F radiochemistry to narrow critical gaps between 19F and 18F chemistry advancements. The review is prepared by leaders in the field, providing an up-to-date and comprehensive overview of C-H labelling strategies and methods with readily available [18F]fluoride. The review is very well-organized, reflecting on the opportunities and challenges of C-H radiolabeling procedures from a radiochemist perspective, clearly focusing on technology dissemination toward clinical translation and clinical application. The authors illustrate different C-H functionalization chemistry with 18F by providing several examples explaining the discussed chemistry."*

Response: We thank the reviewer for these comments.

- *"The provided schemes are critical to following the discussion in the text. A better alignment of the schemes with the text would help to improve readability (E.g. direct sp2 C-H radiolabeling is discussed on page 5, but the corresponding schemes are shown on page 3). Moreover, all schemes should use the same font size and size of presented compounds for better readability."*

Response: We agree with the reviewer that the arrangement of the figures and corresponding text should be changed to improve readability and have rearranged the article accordingly.

- *"The review discusses highly innovative but also complex chemistry using metal and metal-free C-H activation processes. If known, the authors should provide additional information, including schemes on the mechanisms of the discussed C-H radiolabelling method. This would make the review an excellent source of information on the current state-of-the-art C-H labelling strategies with [18F]fluoride and a valuable educational experience on complex reaction mechanisms in the domain of 18F radiochemistry."*

Response: We agree that adding known reaction mechanisms would improve the article. Therefore, we have added information on the reaction mechanisms in current and new schemes, where appropriate.

- "*Overall, this is an excellent review addressing an important research topic. The review is timely and very well written. The research community will highly appreciate the summary of C-H labelling methods with [18F]fluoride.*"

Response: We again thank the reviewer for their positive feedback.

Reviewer 2:

- *"This 'Outlook' describes the current state of C-H radiofluorination methods and details the challenges of these methods in terms of selectivity, precursor stability (and pre-functionalization needs), yield, molar activity, late-stage functionalization, access to densely functionalized substrates, etc. Overall, the topic is well researched and reported with appropriate references and a thorough report on the various C-H radiofluorination methodologies. A few typos and minor suggestions to improve the manuscript are listed below".*

Response: We thank the reviewer for these comments.

- *"The authors are inconsistent with their description of molar activity. In the field, an optimal or high molar activity is considered > 1 Ci per umol. The authors state this parameter in the second page of their manuscript as well. However, in section 2.1 when describing the compound labeled 4- 18F, the authors state it is obtained in high radiochemical yield and Am (the reported Am in Scheme 2a is 0.80 Ci per umol, which is moderate). Again, in the following paragraph, the authors describe compound 5-H and state that radiofluorination of 5-H was*

automated on a microfluidic system in high Am (the reported Am in Scheme 2b is 7.57 mCi per umol, which is very low). Again, in section 2.1.3, in the first paragraph the authors state "4-phenoxybenzene was obtained in 1.37 Ci per umol Am, sufficient for most preclinical studies". This should be considered high Am, the use of the word "sufficient" seems misplaced. Again, in section 2.1.3, in the second paragraph, the authors describe 10-H and state it was "labeled in high RCY and Am" (the Am reported in Scheme 4B is 1.2 Ci per umol and 0.946 Ci per umol, this is lower than the Am obtained for 4-phenoxybenzene for which the authors called "sufficient"). These inconsistencies should be corrected."

Response: We agree that the discussion on A_m was inconsistent and did not accurately reflect the ideal values set out at the beginning of the manuscript. We have altered the language to reflect this accordingly, including the specific examples noted.

- "*In Scheme 3b, there are multiple compounds shown and one yield reported. Is the automated yield reported for the purified 7A-18F (the authors state in the manuscript that automated recoveries refer to isolated material)? As depicted, it looks like there is an inseparable mixture of compounds. Consider revising the figure to clarify the yield is for the purified 7A-18F.*"

Response: This has been clarified in the figure, which now clearly shows the recovery of major product 7A-18F.

- *"In section 3.1.1, the authors describe Scheme 7 in the first paragraph, but it is not referred to in the text. Same is true for the second paragraph of section 3.1.1 and Scheme 8. Please add "Scheme 7" and "Scheme 8" in the appropriate text, when referring to those schemes. In the last paragraph of section 3.1.1, describing compounds 30-32-H, the authors refer to Scheme 8. This is a typo – should be Scheme 9."*

Response: We have ensured that each Scheme is referred to correctly in the text.

- "*For Scheme 2A, I believe the labeling of compound [18F]AC261066 should be written "18F-AC261066" as the brackets are only used when a fluorine atom is being described or is present the name of the compound (which is not the case here). Refer to nomenclature rules detailed in this article: https://www.sciencedirect.com/science/article/pii/S0969805117303189?via%3Dihub*"

Response: We thank for the reviewer for drawing our attention to this figure and the article resource. The compound [18F]AC261066 was labelled correctly, and as intended; however, the structure was incorrect. This has been updated.

- *"In Scheme 2B, it would be better to show the structure of the product, with the CF218F group in the product, in red. The readers can deduce (given the topic of the article and the structure sequences in all the schemes) that the reactant has an 'H' where the 'CF218F' group is located. I think this would be clearer than the current state of Scheme 2B."*

Response: We agree that this would be a clearer representation of this transformation and have made this change.

- *"Lastly, the authors should be careful with the tone of the article when describing their own methodologies versus other groups' reported methodologies. The Sanford and Scott groups have developed numerous, highly valuable methods and many are described in this manuscript. In an Outlook such as this, it is critical to describe each method with a non-bias eye. I would suggest the authors review their manuscript with this mind. For example, in the first paragraph of section 2.2.1, the authors describe their own methodology and state . . . "facilitates excellent para-regioselectivity", "with excellent chemoselectivity", "carries*

excellent prospects". In one paragraph, the word 'excellent' is used three times. . . consider revising."

Response: We appreciate the reviewer pointing this out. We have altered some of the language, including this specific example, with these comments in mind.

oc-2024-009976.R2

Name: Peer Review Information for "C–H Labeling with [¹⁸F]Fluoride: An Emerging Methodology in Radiochemistry"

Second Round of Reviewer Comments

Reviewer: 2

Comments to the Author
all concerns have been addressed

Reviewer: 1

Comments to the Author
The authors addressed the reviewers' comments. The article should be published in the current form.

Author's Response to Peer Review Comments:

We have reduced the length of the synopsis as requested... no other changes have been made.
